# Squamous cell carcinoma of the tongue in a female with advanced breast cancer: A case report of an elderly patient presenting with two types of cancer

**DOI:** 10.3892/ol.2014.2097

**Published:** 2014-04-28

**Authors:** RAFFAELE ADDEO, ALBERTO NAPOLITANO, LILIANA MONTELLA, FILIPPO RICCIARDIELLO

**Affiliations:** 1 Medical Oncology Unit, The San Giovanni di Dio Hospital, Frattamaggiore, Naples 80027, Italy; 2 ORL Unit, AORN Cardarelli, Naples 80100, Italy; 3 Department of Clinical Otology, ENT Clinic, ‘Federico II’ University, Naples 80131, Italy

**Keywords:** breast cancer, squamous cell carcinoma, chemotherapy, immunosuppression

## Abstract

Elderly patients with cancer are frequently undertreated as they are considered to be unfit for treatment due to inaccurate estimations of the operative risk. In the current study, the case of an 81-year-old female smoker with advanced breast cancer is presented. The patient had received numerous cycles of chemo- and hormonal therapy and the cancer only progressed locally. After six years, the patient developed a second type of cancer; a moderately differentiated squamous cell carcinoma of the tongue. The patient refused any local treatment and she received supportive care only. There is currently a lack of data regarding the molecular mechanisms of second primary cancers as well as other delayed outcomes following cancer treatment. Therefore, it is proposed that chemotherapy may promote the presentation of the second cancer as a result of immunosuppression.

## Introduction

The incidence of breast cancer is higher in elderly patients (>65 years) when compared with younger patients (<65 years old), with the elderly being associated with an increased mortality risk (1). For elderly patients, age-related factors must be considered when prescribing the cancer treatment and supportive care. These factors include organ function, comorbidity, consumption of other medication and cognitive function. In this type of patient, quality of life remains a primary objective for the clinician. In the last decade, a large quantity of research has provided support for the importance and efficacy of chemotherapy for the treatment of elderly patients with metastatic breast cancer (2).

However, previous data has implicated the role of chemotherapy or radiotherapy in the development of second cancers following adult or pediatric cancer (3). The delayed effects of treatment may be modified by moderate or low-penetrance genetic traits or by additional gene-environment and gene-gene interactions. As variations in DNA repair genes appear to have a role in the susceptibility to *de novo* cancer (4), it is likely that these variations modify cancer risk following exposure to DNA-damaging agents, including radio- and chemotherapeutic agents.

As yet, the identification of patient subgroups that may possess a heightened susceptibility to developing cancer or other adverse sequelae has not been systematically addressed. However, an increase in cases of head and neck cancer (HNC) has been observed in the elderly (5).

The development of squamous cell carcinoma of the tongue may depend on various factors, which include the human papilloma virus (HPV), alcohol consumption and smoking (6). In the present study we describe an elderly patient who presented with advanced breast cancer that was intensively treated, who subsequently developed a second cancer of the tongue. Patient provided written informed consent.

## Case report

**Figure 1:**
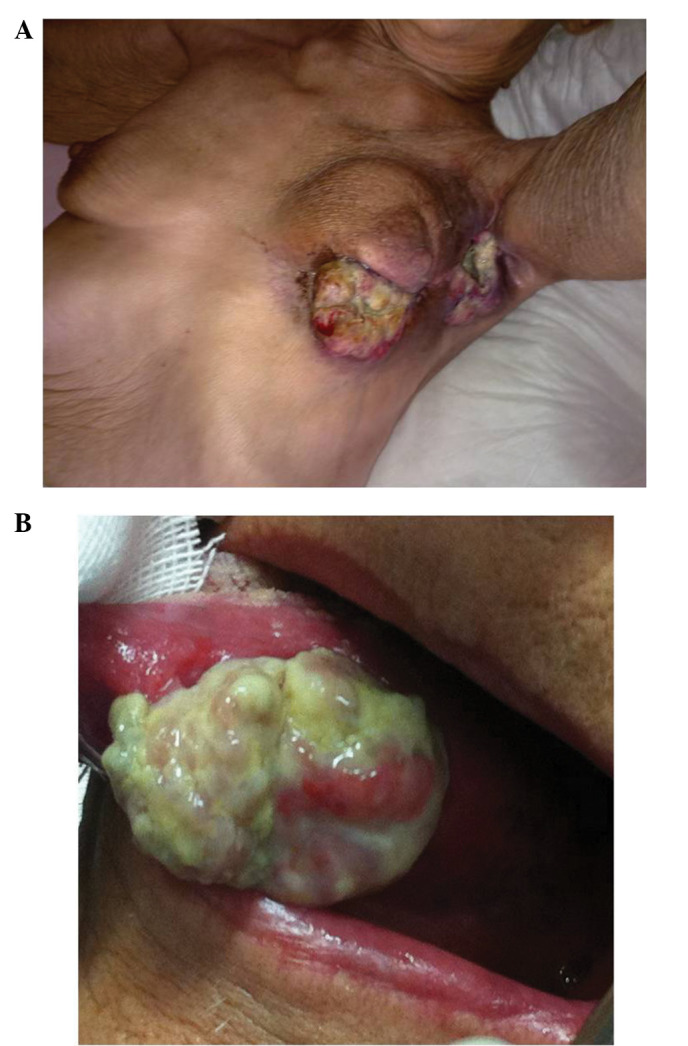
(A) The left breast is predominated by the breast cancer. Furthermore, the image demonstrates the spread of cancer to the left axilla. (B) An ulcerovegetative lesion involving the floor of the mouth and the left border of the tongue.

In March 2005, an 81-year-old female smoker was diagnosed at the Medical Oncology Unit of the San Giovanni di Dio Hospital (Frattamaggiore, Italy) with advanced left breast cancer. The tumor was characterized as estrogen and progesterone receptor-positive, and human epidermal growth factor 2-positive. The tumor was treated with numerous cycles of chemo- and hormonal therapy and the cancer only progressed locally. The patient did not show metastases to any sites during the follow-up period. The patient received the following combination treatments: Capecitabine, anastrozole, pegylated liposomal doxorubicin and vinorelbine; trastuzumab and fulvestrant; docetaxel, gemcitabine and trastuzumab; and capecitabine and lapatinib. In August 2011, the patient presented with a painful nodule on the tongue, which had been gradually increasing in size. The patient’s clinical analysis identified an elevated leukocyte count (19.32×10^3^/μl in September 2011, with the level of neutrophils above the normal range). This tumor progressed to involve the floor of the mouth and the left border of the tongue. The histological analysis determined that is had not originated as a result of metastasis from the breast lesion; it was diagnosed as a moderately differentiated squamous cell carcinoma. The patient refused any local treatment and received only supportive care. The involvement of the left breast and axilla by the breast cancer, and the tongue cancer are demonstrated in Fig. 1A and B, respectively. The breast cancer, despite its advanced status, was less complex to manage in the elderly patient compared with the management of the tongue tumor. The patient succumbed to the tongue cancer as a result of bleeding, the associated anemia, and increasing problems with food intake and cancer cachexia.

## Discussion

Tobacco smoking and alcohol consumption are the principal risk factors for developing tongue squamous cell carcinoma (7). In addition, chewing tobacco, radiation exposure, HPV, as well as immune deficiencies have been implicated (8). The prognosis for patients presenting with tongue cancer varies depending on the stage and site.

The percentage of elderly people with HNC is rising as a result of the increasing average lifespan; ~10% of HNC occur in patients aged ≥80 years (5). Management of this subpopulation has become a source of debate due to the paucity of randomized data regarding the effect of age on treatment response as well as morbidity associated with the treatment of HNC. Furthermore, approximately two-thirds of HNC patients present with locoregionally advanced stage (III and IV) disease§. This requires multimodality therapy, including surgery, radiation and/or chemotherapy. Elderly patients may not be suitable for aggressive multimodality treatment due to their increased comorbidity status, poor treatment tolerance and the toxicity of treatments, such as radiotherapy and chemotherapy. The Health-Related Quality of Life assessment is currently considered to be one of the primary objectives of clinical research concerning this subpopulation of patients (9).

Decision-making in the present case was based on the patient’s characteristics and age, however, was also based on a performance status evaluation, comorbidity and a patient-reported outcomes assessment. This mode of conduct is similar to that which was proposed in a recent study regarding geriatric patients with HNC (10).

An additional case of a young female who presented with tongue cancer that later developed into breast cancer was identified in the literature (11). In addition, the quantity of patients presenting with two types of cancer, with oral cancer as the primary malignancy, is reportedly on the rise (12).

In conclusion, in the present case, the origin of the second cancer was unknown, however, the patient was an elderly female who smoked, which may have been a possible cause of the tongue tumor. Furthermore, previous chemotherapeutic treatment may have been involved in predisposing the patient to develop the second neoplasm. A possible mechanism is chemotherapy-induced immunosuppression, which is a well-recognized cause of skin tumors, specifically squamous cell carcinomas (13). The present case highlights the influence of individual risk factors and chemotherapy-induced immunosuppression in the selection of treatment for elderly cancer patients to prevent the occurrence of a second cancer.
